# Whey Protein Hydrolysate and Pumpkin Pectin as Nutraceutical and Prebiotic Components in a Functional Mousse with Antihypertensive and Bifidogenic Properties

**DOI:** 10.3390/nu11122930

**Published:** 2019-12-03

**Authors:** Evgeniya Yu. Agarkova, Alexandr G. Kruchinin, Olga A. Glazunova, Tatyana V. Fedorova

**Affiliations:** 1 All-Russian Research Institute of Dairy Industry, Moscow 115093, Russia; kruchinin-vnimi@yandex.ru; 2 A. N. Bach Institute of Biochemistry, Research Centre of Biotechnology of the Russian Academy of Sciences, Moscow 119071, Russia

**Keywords:** whey protein hydrolysates, pumpkin pectin, mousse, functional food, antioxidant activity, hypotensive properties

## Abstract

Systematical consumption of functional products has a significant positive effect on health and can reduce the risk of diseases. The aim of this study was to investigate the possibility of using whey protein hydrolysate (WPH) and pumpkin pectin as ingredients in a functional mousse, to evaluate the mousse’s antioxidant and hypotensive activities in vitro, and to evaluate the effect of the long-term intake of mousse samples on the progression of hypertension in spontaneously hypertensive rats (SHRs) and on the microbiome status in Wistar rats with antibiotic-induced dysbiosis. The experimental mousse’s in vitro antioxidant activity (oxygen radical absorbance capacity) increased by 1.2 times. The hypotensive (angiotensin-1-converting enzyme inhibitory) activity increased by 6 times in comparison with a commercial mousse. Moreover, the addition of pectin allowed the elimination of the bitter aftertaste of WPH. In vivo testing confirmed the hypotensive properties of the experimental mousse. The systolic blood pressure in SHRs decreased by 18 mmHg and diastolic blood pressure by 12 mmHg. The experimental mousse also showed a pronounced bifidogenic effect. The *Bifidobacterium* spp. population increased by 3.7 times in rats orally administered with the experimental mousse. The results of these studies confirm that WPH and pumpkin pectin are prospective ingredients for the development of functional mousses.

## 1. Introduction

Beyond doubt, the almost 2500-year-old quote of the great Hippocrates (Hippocrates of Kos, 460–370 BC): “Let food be your medicine and let your medicine be your food”, can be considered as a motto of modern food science. Nowadays, the corpus of epidemiological evidence linking different diseases, such as cardiovascular disease, obesity, hypertension, diabetes, and even cancer, to dietary factors is steadily growing. Many scientists consider proper diet as the main factor for reduction of the risk and limitation of the progression of many chronic diseases [1]. Increased awareness about the relation between food and health has resulted in the emergence of new terms: “functional food”, “nutraceuticals” and “prebiotics” [2]. While functional food is a kind of food that can provide health benefits beyond those of basic nutrition [3], nutraceuticals are health-promoting compounds isolated or purified from food sources [4,5]. The term “prebiotic” is used to refer to food compounds that promote the growth of health-beneficial microorganisms of the intestinal microbiome.

Among the different types of nutraceuticals, peptide nutraceuticals are the most versatile ones [6]. Typically, bioactive peptides are short chains of three to twenty amino acids linked together by peptide bonds. The main source of nutraceutical peptides is the enzymatic digestion of dietary proteins during their gastrointestinal transit or in the process of food fermentation [7]. Although the precise molecular mechanism of action for many bioactive peptides remains to be discovered, the number of different proven nutraceutical properties that they can possess is truly enormous. Over the past decade, the potential of bioactive peptides to reduce the risk of chronic diseases and to promote human health has stimulated ever-increasing scientific and commercial interest; nowadays, different protein-peptide hydrolysates are incorporated in many food products, including those with functional properties [8,9,10].

Whey is a watery, poor in casein part of milk remaining after the formation of curd. Whey proteins (mainly β-lactoglobulins and lactalbumins) are considered as a rich source of bioactive peptides [8,11]. It was demonstrated that the hydrolysis of whey proteins can produce peptides with such nutraceutical properties as antioxidant, antihypertensive, antithrombotic, anti-inflammatory, antimicrobial, anticancer, immunomodulatory etc. [8,12,13,14]. One of the most interesting groups of peptides that can be found in whey protein hydrolysates (WPHs) is those that inhibit angiotensin-converting enzyme (EC 3.4.15.1, ACE). The main function of this enzyme is to convert the angiotensin I decapeptide (officially called proangiotensin) into the active vasoconstrictor hormone—angiotensin II, through the removal of two C-terminal amino acid residues. Hence, the enzymatic action of ACE leads to the constriction of blood vessels and, consequently, to an increase in blood pressure. The use of ACE-inhibiting peptides from WPHs offers a new nutritionally based way of management and, possibly, treatment for persons with essential (primary) hypertension [15,16]. In contrast to the standard chemically based medications, such an approach will have less undesirable harmful side effects (adverse drug reactions).

Generally, two main problems associated with the inclusion of bioactive peptides into a product’s composition have to be considered: (1) since the inclusion of peptides can significantly alter the taste and texture of the final product, some additional alternation of the original product formulation has to be considered to alleviate this problem; (2) after the inclusion into the product, the peptides’ bioavailability and, consequently, nutraceutical effects can change significantly; hence, testing of the product itself rather than preliminary assessment of the added peptides is of crucial importance.

As it is well established, the gut microbiome is a crucial component of the human organism, and systematic consumption of prebiotics can drastically improve health and substantially prevent the possibility of disease. Pectin is an acidic heteropolysaccharide (rich in galacturonic acid) with well-known prebiotic properties [17,18]. Currently, pectin, mainly derived from citrus peels, is widely used in the food industry as a gelling agent, stabilizer and fat replacer. Moreover, exhibiting antioxidative, immunomodulatory, cytoprotective, hypocholesterolemic, hypoglycemic, prebiotic and other activities [17,19,20,21], pectin from different sources provides a promising basis for manufacturing of a wide range of functional foods.

Currently, ready-to-eat dairy desserts with different tastes, flavors and textures represent a big segment of the global market. Aside from the nutritional quality of such desserts, they could be turned into functional foods by incorporation of different ingredients, e.g., bioactive peptides and prebiotic dietary fibers [22,23]. From a wide spectrum of produced dairy desserts, mousses have emerged as an attractive system for incorporation and testing of the effects of different functional ingredients [24].

The goal of the current work was to develop a formulation of mousse that contains protein-peptide hydrolysate from cheese whey proteins and pumpkin pectin. The final product had to demonstrate sufficient antihypertensive activity, while possessing good prebiotic properties and adequate organoleptic characteristics.

## 2. Materials and Methods

### 2.1. Hydrolysis of Cheese Whey

Enzymatic hydrolysis of whey from hard Montasio cheese was conducted under the previously selected conditions [25]: Protamex (Novozymes A/S, Bagsværd, Denmark) and Alcalase (Novozymes A/S, Bagsværd, Denmark) enzyme preparations (3:1 ratio, wt% of the protein content), 90 min reaction time, pH 7.0, and a temperature of 50 °C. The degree of hydrolysis was 12%.

### 2.2. Preparation of Mousse Samples

Based on a commercial recipe—Russian Technical Regulations TU 9222-021-00419785-2016 (“VNIMI”, Russia)—two series of mousse products were manufactured. The products in series I—reference products—were gelled by the addition of guar gum (traditional method) (Table 1). The samples of series II were gelled with the addition of guar gum and pumpkin pectin in a 1:1 ratio (Table 1). For each series, mousse samples with 0%, 25%, 50%, 75% and 100% replacement of cow’s milk by WPH were manufactured.

The process of extraction and characterization of pumpkin pectin was previously described in [26].

In brief, the product preparation was performed as follows: (1) cow’s milk, cream and WPH were mixed together, and low fat quark, skimmed milk powder and sugar were dissolved in the mixture by stirring; (2) the mixture was placed in a water bath and heated up to 40 °C; (3) the stabilizers (guar gum, pectin, gelatin) and the acidity regulator were blended in with a kitchen blender, 4200 MQ9087X (Braun, Kronberg, Germany), at 500 rpm for up to 5 min; (4) the mixture was heated up with agitation to 75 °C and incubated for 5 min; (5) the mixture was cooled down in an ice bath to 20 °C and whipped by the kitchen mixer E700, equipped with a wire whip (Bork, Moscow, Russia), at 1500 rpm for 5 min.

The protein content of the samples was determined by Kjeldal’s method (conversion factor 6.25) using Kjeltec 8200 (FOSS, Höganäs, Sweden).

### 2.3. In Vitro Bioassays

For the in vitro measurement of antioxidant and hypotensive activities, the mousse samples were mixed with distilled water, and the mixture was homogenized using Silent Crusher S (Heidolph, Schwabach, Germany) with 7F head (Heidolph, Schwabach, Germany) at 60,000 rpm for 3 min.

#### 2.3.1. Antioxidant Activity Assay

The in vitro antioxidant activity in the mousse samples was determined by the ORAC (Oxygen Radical Absorbance Capacity) fluorescence method [27] using a Synergy 2 microplate photometer–fluorometer (BioTek, Winooski, VT, USA). The peroxyl radical was generated directly in the reaction medium during the thermal decomposition of the azo compound 2,2′-azobis (2-methylpropionamidine) dihydrochloride (AAPH, Sigma-Aldrich, St. Louis, MO, USA), initiated by incubation at 37 °C for 10 min according to [28]. The antioxidant activity was expressed as the amount of Trolox (Sigma-Aldrich, St. Louis, MO, USA) molar equivalents (TE, μM) per g of protein.

#### 2.3.2. Angiotensin Converting Enzyme Inhibition Assay

The in vitro angiotensin converting enzyme inhibition (ACEI) in the mousse samples was determined by their ability to inhibit angiotensin I-converting enzyme (Sigma-Aldrich, St. Louis, MO, USA). o-Aminobenzoyl-Phe-Arg-Lys(dinitrophenyl)-Pro (Sigma-Aldrich, St. Louis, MO, USA) was used as a substrate with internal fluorescence quenching [29]. The measurements were carried out on a Synergy 2 microplate photometer–fluorometer (BioTek, Winooski, VT, USA). 

To compare the hypotensive properties of mousses, the concentration IC_50_ was determined at which ACE activity decreased by 50%. IC_50_ was expressed as (mg of protein)/mL.

### 2.4. Animal Studies

The maintenance, feeding, and care of animals, as well as all experimental manipulations and removal from the experiment, were carried out in accordance with the directive of the Ministry of Health of Russia, dated 1 April 2016, N199n “On the Approval of the Rules for Good Laboratory Practice” and the “International Rules for the Humane Treatment of Animals” of the Directive of the European Parliament and the Council of the European Union 2010/63/EC, dated September 22, 2010, “On the protection of animals used for scientific purposes”. The experimental protocol No. 12 of 6 August 2018 was approved by the Ethics Committees for Animal Research of the Federal Research Center Fundamentals of Biotechnology of the Russian Academy of Sciences. All surgeries were performed under anesthesia (carbon dioxide gas), and all efforts were made to avoid suffering.

During the experiments, all rats consumed a standard diet (Laboratormkorm, Moscow, Russia) *ad libitum*.

For the in vivo study of the hypotensive and bifidogenic properties, the experimental samples were orally administered by gastric intubation for 45 and 14 days, respectively. Before the administration, the samples were dissolved in drinking water. The final dose of the products administrated to the rats was 2 g of product per 1 kg of body weight (i.e., 200 mg of protein per 1 kg of body weight).

The procedure of euthanasia was performed as follows: 12 h before the end of the experiment, all feed residues were removed from the cells; upon the completion of the experiment, the experimental animals were placed in a chamber for carbon dioxide euthanasia (VetTech, Congleton, UK) for 3–5 min, depending on the body weight of the animal. The flow rate of the carbon dioxide rotameter was set to 3.5 dm³/min. The end of the exposure time of the animal in the chamber for carbon dioxide euthanasia was established visually by cessation of respiratory movements.

#### 2.4.1. Assay of Antihypertensive Effect in Spontaneously Hypertensive Rats (SHRs)

The in vivo study of the hypotensive properties of mousse samples was performed on 14-week-old male spontaneously hypertensive rats (SHRs) [30] (Puschino Kennel of Laboratory Animals, Pushchino, Russia). At the beginning of the experiment, the rats’ body weight was 280 ± 20 g. The systolic blood pressure (SBP) and the diastolic blood pressure (DBP) were 184 ± 15 mmHg and 112 ± 14 mmHg, respectively, which corresponds to the typical SBP and DBP in SHRs of this age group [31].

For the experiment, the animals were randomly divided into three groups of ten animals each: (1) receiving 2 mL of distilled water (the control group); (2) receiving 2 mL of diluted commercial mousse; (3) receiving 2 mL of diluted experimental mousse—100% replacement of cow’s milk by WPH, 50:50 guar gum: pumpkin pectin.

The blood pressure of the rats was measured by the tail-cuff method with a Coda Monitor (Kent Scientific, Torrington, CT, USA) with a set of cuffs and sensors, RAT-CUFFKIT (Kent Scientific, Torrington, CT, USA). At least ten measurement cycles were performed for each animal, and the results were averaged.

On the 45th day of the experiment, the animals were euthanized and blood was collected. The blood serum was separated via centrifugation for 10 min with an Eppendorf 5702R centrifuge (Eppendorf, Hamburg, Germany) at 4 °C and 2000 *g*. The concentration of angiotensin I and angiotensin II in the blood serum was determined using Angiotensin I and Angiotensin II ELISA kits (Enzo, Farmingdale, NY, USA).

#### 2.4.2. Assay of Bifidogenic Effect in Wistar Rats

The bifidogenic properties of mousse samples were studied on male Wistar rats (the same breed from the vivarium of the Research Centre of Biotechnology RAS (Moscow, Russia)) with an antibiotic-induced dysbiosis [32]. At the beginning of the experiment, the rats’ body weight was 410 ± 30 g.

The dysbiosis was induced by daily intragastric administration of the antibiotic fluoroquinalone baytril (10%) (Baytril^®^, Bayer, Leverkusen, Germany) at a dose of 10 mg per 1 kg of animal body weight. Introduction of the antibiotic was performed 30 min before the sample administration.

For the experiment, animals were randomly divided into four groups of ten animals each: (1) receiving 2 mL of distilled water without antibiotic (intact-control group); (2) receiving 2 mL of distilled water and antibiotic; (3) receiving 2 mL of diluted commercial mousse and antibiotic; (4) receiving 2 mL of diluted experimental mousse—100% replacement of cow’s milk by WPH, 50:50 guar gum: pumpkin pectin—and antibiotic.

On the 14th day of the experiment, the animals were euthanized and fecal samples were collected from the colon.

To quantify the lactobacteria and bifidobacteria, 1.0 g of rat feces was added to 50 mL of sterile 0.9% sodium chloride solution. Serial dilutions were prepared, and the inoculations were made into selective growth media for *Lactobacillus* spp. (MRS medium, 72 h, 37 °C) and *Bifidobacterium* spp. (TOS-MUP agar medium, 72–120 h, 37 °C).

### 2.5. Sensory Evaluation

All products were stored for 1 day at 5 °C before the sensory evaluation. The sensory evaluation was carried out using eight panelists familiar with the product. The samples were evaluated for consistency, flavor, taste, bitterness, color and overall acceptability, on a five-point hedonic scale.

### 2.6. Statistical Analysis

The results were analyzed by ANOVA; the diagnostic for normality and homogeneity of variance assumptions was performed by visual investigation of Normal Probability and Residual plots after model fitting. All the ANOVAs were followed by Tukey’s HSD (honestly significant difference) post hoc tests (*p* < 0.05). Whenever appropriate, the data are represented by the mean ± standard deviation (SD).

## 3. Results and Discussion

### 3.1. In Vitro Antioxidant and ACEI Activities of Mousse Samples

At the first stage of the study, five samples of mousses were prepared. One sample was prepared according to a commercial recipe, and the other four with the replacement of 25%, 50%, 75% and 100% of cow’s milk by a Montasio cheese WPH. In our previous studies [25], the composition of this whey was characterized (19% α-lactalbumin, 56% β-lactoglobulin and 11% κ-casein), and the presence of peptides with potential antioxidant and ACEI activities in its hydrolysate was demonstrated.

**Figure 1:**
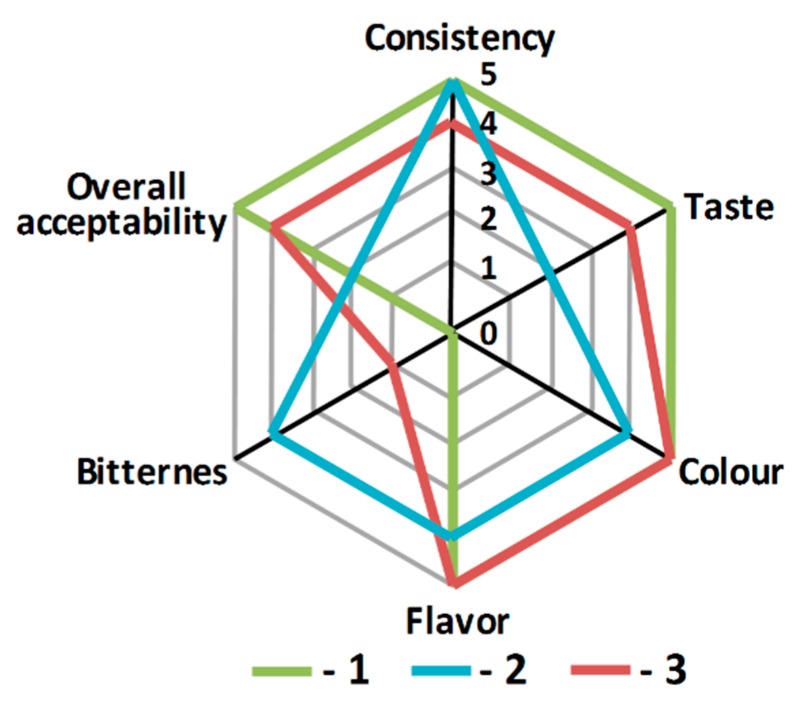
Organoleptic evaluation of the mousse samples. 1—commercial mousse, 2—experimental mousse with 100% WPH and 100% guar gum, 3—experimental mousse with 100% WPH, 50% guar gum and 50% pectin.

As a result, a positive correlation between the amount of WPH in the product and the product’s antioxidant and ACEI activities was demonstrated—the higher the hydrolysate content, the better the antioxidant and hypotensive activities. For the mousse sample in which all the cow’s milk was replaced by whey hydrolysate, the antioxidant activity increased by 1.2 times, and the ACEI activity by 2.5 times (Table 2). Unfortunately, upon the introduction of hydrolysate, the product’s organoleptic attributes deteriorated, with the appearance of a less pleasant texture and a bitter aftertaste (Figure 1).

It is known that many hydrolysates of plant and animal proteins often have a bitter aftertaste. Detailed investigation of this phenomenon revealed that the main reason for hydrolysate bitterness is low molecular mass peptides containing hydrophobic amino acid residues [33]. Additionally, one work [34] demonstrated that the bitter taste of peptides is enhanced by a proline residue at their center. It should be noted that the inhibition of ACE is performed by hydrophobic peptides that exert high affinity toward the active site of ACE [35]. In our previous study [25], it was shown that most of the proposed hypotensive peptides found in the hydrolyzed whey from Montasio cheese contain hydrophobic amino acid residues, including proline in the central position. Previously, Zeeb et al. [36] reported that pectin can reduce the bitter taste of plant-derived proteins. Also, it was shown that mixed gels containing different polysaccharides could have an improved structure [37,38]. Hence, at the second stage of the study, guar gum in the original recipe was replaced by highly methoxylated pumpkin pectin [26]. Model systems containing WPH and different ratios of guar gum and pumpkin pectin (30:70, 50:50 and 70:30 w/w) were preliminarily investigated. The best ACEI activity was observed for the sample with pumpkin pectin and guar gum in the ratio of 50:50 (data not shown). 

The introduction of pumpkin pectin with guar gum in the ratio of 50:50 (w/w) into the mousse formulation led to an improvement in the perceived sensorial acceptance of the product; moreover, the bitter taste almost completely disappeared (Figure 1). As can be seen from Table 2, similarly with the pectin-free products, both the antioxidant and ACEI activities of the pectin-containing products increased with an increase in their WPH content. However, the ACEI activity in the pectin-containing product with the complete replacement of milk by WPH was 2.8 times higher than in the product without pectin, and antioxidant activity was slightly reduced. The change in the biological activity of mousses after adding pectin to the formulation can be explained by the different specificity of the interaction of guar gum and pectin with biologically active peptides, since these polysaccharides have a different structure.

Thus, it was shown that the product containing both WPH and pumpkin pectin has acceptable organoleptic properties and at the same time has a pronounced in vitro ACEI activity in comparison to the commercial sample.

### 3.2. In Vivo Hypotensive Properties of Mousse Samples

Although in vitro studies are the conventional first step in the evaluation of potential antihypertensive properties of products, further in vivo studies are always required for the definitive conclusion about the product’s true biological effects. One of the most useful animal models currently used to evaluate the antihypertensive effect in vivo is SHRs. For many years, SHRs were used as an animal model of essential (primary) hypertension and cardiovascular diseases [31].

In this study, the effect of long-term administration of the samples of experimental and commercial mousses on blood pressure in SHRs was evaluated. As an experimental sample of mousse, the sample that demonstrated the highest ACEI activity in the in vitro experiments—the recipe with complete replacement of cow’s milk with WPH and the addition of pumpkin pectin—was selected. The control group of animals received water instead of mousse samples. The design of the experiment was a classical pretest-posttest comparison group design, which was analyzed by the one-way ANOVA omnibus test on the gain scores (i.e., difference between after-treatment scores and before-treatment scores) followed up by the Tukey’s HSD test.

**Figure 2:**
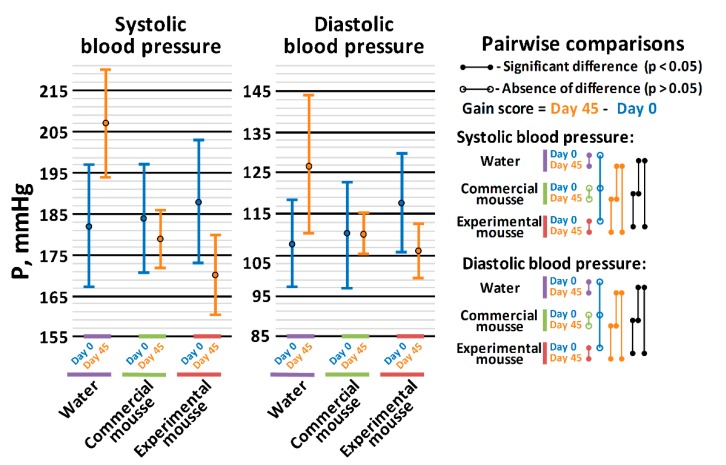
Changes in systolic and diastolic blood pressure in SHRs (spontaneously hypertensive rats) after 45 days of oral administration of water (control), commercial and experimental mousse samples.

The result of ANOVA suggested the presence of a significant difference (*p* < 0.05) between the gain scores for both systolic blood pressure (SBP) and diastolic blood pressure (DBP) in rats. The *post hoc* Tukey’s HSD test (*p* < 0.05) showed that after the 45 days of administration: in SHRs treated with water, both SBP and DBP statistically significantly increased (from 181 ± 16 and 108 ± 11 mmHg to 207 ± 13 and 127 ± 17 mmHg, respectively); in SHRs treated with the commercial mousse sample, both SBP and DBP were stabilized at their initial levels; and in SHRs treated with the experimental mousse sample, both SBP and DBP significantly decreased (from 188 ± 15 and 118 ± 12 mmHg to 170 ± 16 and 106 ± 7 mmHg, respectively) (Figure 2). Thus, the experimental mousse sample, containing WPH and pumpkin pectin, exhibited a more pronounced antihypertensive effect than the commercial mousse sample. These data correlate with the ACEI activity of the mousse samples in vitro. The reduction of SBP by 18 mmHg in SHRs receiving an experimental mousse sample is comparable with the effect of fermented milk products supplemented with hypotensive peptides [39].

**Figure 3:**
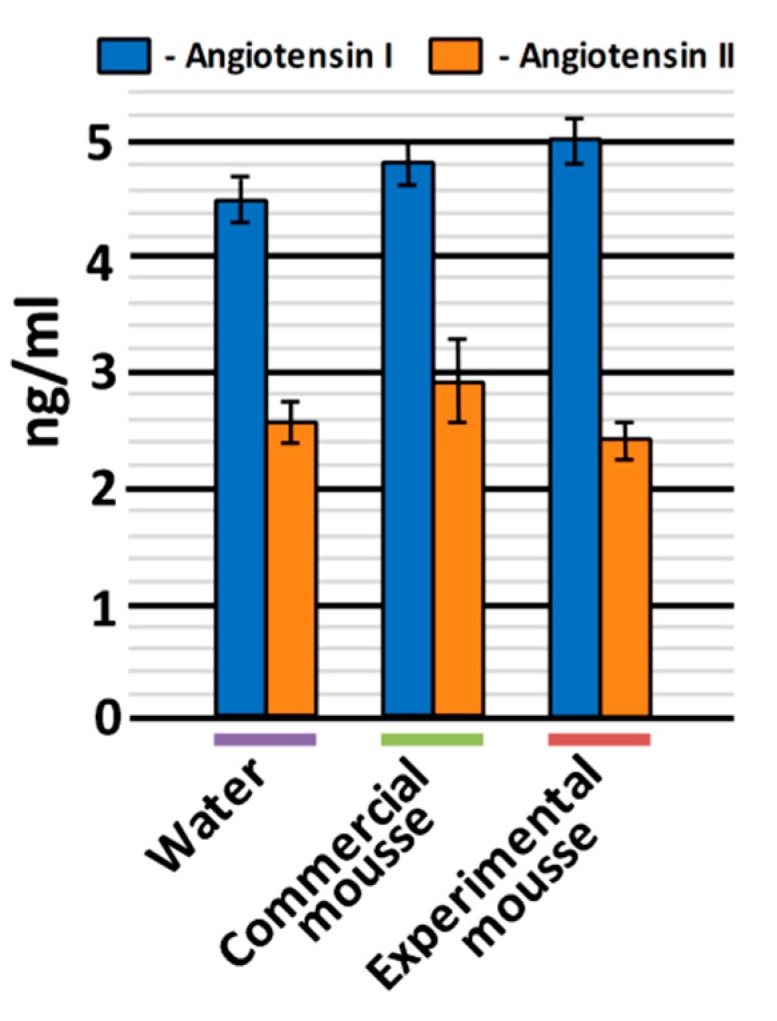
Angiotensin I and angiotensin II concentrations in blood serum of SHRs after 45 days of oral administration of water (control), commercial and experimental mousse samples.

As it was briefly mentioned in the Introduction, one of the possible mechanisms responsible for the antihypertensive effect of bioactive peptides is ACE inhibition, although other (including currently undiscovered) mechanisms are possible [40]. To explore whether ACE inhibition really took place in SHRs receiving the mousse samples, the concentrations of angiotensin I and angiotensin II were measured in rats’ blood serum after 45 days of the experiment (Figure 3).

For angiotensin I, the concentration was the lowest in the blood of animals treated with water (4.47 ± 0.21 ng/mL) and the highest in the blood of animals treated with the experimental mousse sample (5.00 ± 0.20 ng/mL) (Figure 3). At the same time, the concentration of angiotensin II in the blood of animals treated with the experimental mousse was lower (2.46 ± 0.15 ng/mL) than in the blood of animals treated with water and the commercial mousse sample (2.59 ± 0.23 and 2.91 ± 0.33 ng/mL).

The obtained data regarding concentrations of angiotensin I and angiotensin II in the blood of SHRs are in agreement with the data about SBP and DBP (Figure 2). Indeed, since ACE is responsible for the conversion of angiotensin I to angiotensin II [41], a strong vasoconstrictor agent, it is expectable that, in the case of inhibition of this enzyme, the concentration of its substrate will increase and of its product, will decrease. Hence, the presented results indirectly support ACE inhibition as an in vivo mechanism for the antihypertensive effect of long-term oral administration of the tested mousse sample containing WPH and pumpkin pectin.

### 3.3. Prebiotic Effect In Vivo

**Figure 4:**
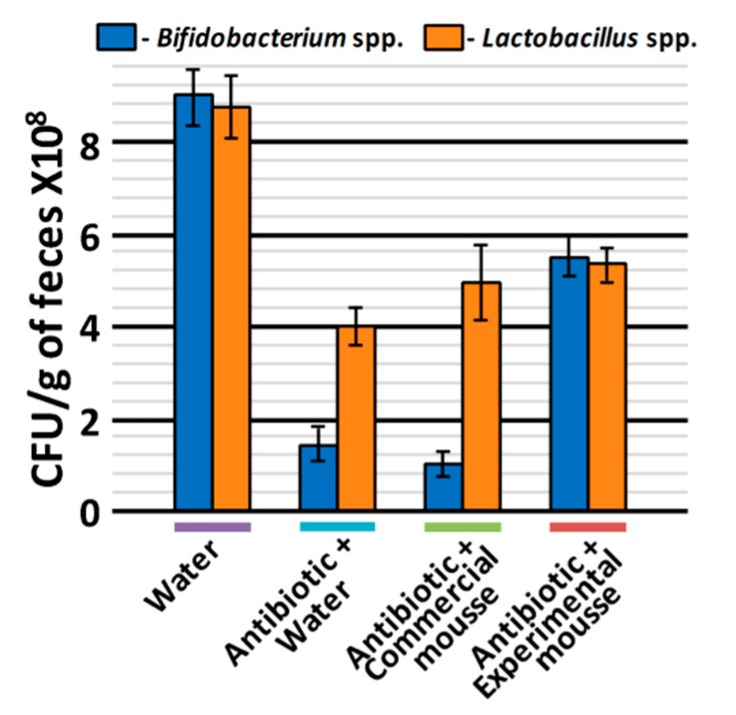
The number of viable *Bifidobacterium* and *Lactobacillus* spp. in the feces of Wistar rats with antibiotic-induced dysbiosis, measured after 14 days of oral administration of water (negative-control), commercial and experimental mousse samples. Rats without antibiotic treatment were used as an intact control.

The prebiotic effect of commercial and experimental mousse samples was studied in male Wistar rats with antibiotic-induced dysbiosis; rats without antibiotic treatment were used as an intact control. After antibiotic treatment, the negative-control group of rats was administered with only water, while the two experimental groups—with commercial and experimental mousse samples, respectively. Antibiotic treatment resulted in a moderate dysbiosis that manifested as a decrease in *Lactobacillus* spp. and *Bifidobacterium* spp. populations in the rat gut microbiome. Compared to the intact control group, the number of *Lactobacillus* spp. in the feces of rats administered with water decreased by 2.2 times and *Bifidobacterium* spp.—by 9 times (*p* < 0.01) (Figure 4). Both commercial and experimental mousse samples had a similar in size positive effect on the number of *Lactobacillus* spp. —(5.0 ± 0.7) × 10^8^ and (5.6 ± 0.4) × 10^8^ CFU/(g of feces) instead of (4.0 ± 0.4) × 10^8^ CFU/(g of feces) in the negative-control group. At the same time, the *Bifidobacterium* spp. population increased in the group administered with the experimental mousse sample by 3.7 times (compared to the negative-control), while in the group administered with the commercial mousse sample, the *Bifidobacterium* spp. population was even lower than in the group administered with water (Figure 4). Thus, a prominent bifidogenic effect was shown in Wistar rats treated with the mousse sample containing WPH and pumpkin pectin.

The bifidogenic effect of the experimental mousse sample could be explained by the presence of both WPH and pectin in its formulation. Although the mechanism underlying the bifidogenic effect of protein-peptide hydrolysates is still unclear, it was shown that milk protein hydrolysates improve the growth of bifidobacteria [42,43]. Peptides from hydrolysates could provide an additional nutrition source for bifidobacteria, which have a weak proteolytic system [44,45]. Furthermore, the specific action of individual milk-derived peptides on bifidobacteria growth was demonstrated [46,47].

Pectin is a soluble indigestible dietary fiber. Like other dietary fibers, pectin reaches the large intestine almost intact, where it is fermented by the gut microbiota [48]. Pectin and pectin-derived oligosaccharides have been identified as prebiotics that promote growth of *Bifidobacterium* and *Lactobacillus* spp. [49,50,51].

## 4. Conclusions

In the current work, we developed an antihypertensive and bifidogenic functional product (mousse) containing WPH and pumpkin pectin. The developed product inhibited ACE activity in vitro and exerted an antihypertensive effect in vivo. The mousse treatment reversed the development of hypertension in SHRs. After oral administration for 45 days, SBP was reduced by 18 mmHg, and DBP was reduced by 12 mmHg. Moreover, it was demonstrated that the mousse treatment increased the concentration of angiotensin I and decreased the concentration of angiotensin II in rats’ blood serum. Hence, the observed in vitro ACEI effect was confirmed by in vivo trials, supporting the ACEI mechanism of the antihypertensive effect.

While the addition of WPH supplied bioactive peptides that, possessing ACEI activity, provided the main antihypertensive functionality to the product, the addition of the pumpkin pectin to the final product in fact “killed two birds with one stone”. The pectin not only mitigated the bitterness of WPH, but also brought additional prebiotic properties to the final product. Moreover, to the best of our knowledge, this is the first report that demonstrates the possibility of mitigating protein-peptide hydrolysates’ bitterness by addition of pectin.

In conclusion, we demonstrated that WPH and pumpkin pectin could be successfully incorporated in a dairy mousse, a promising functional product for prevention and maintenance therapy of essential hypertension and dysbiosis. However, further extended clinical trials in human volunteers are necessary for investigating the efficiency and safety of the proposed functional product in the prevention of hypertension as well as the treatment of hypertension.

## Figures and Tables

**Table 1:** Composition of mousse samples.

Ingredient	Amount (g/100 g of Product)	
	Series I	Series II
Low fat quark	40.0	40.0
Cream (10% fat)	11.65	11.65
Cow’s milk (2.5% fat)	34.14	34.14
Sugar	10.7	10.7
Skimmed milk powder	2.0	2.0
Gelatin	0.83	0.83
Guar gum	0.42	0.21
Pumpkin pectin	-	0.21
Acidity regulator	0.26	0.26

All preparations were stored at 5 °C until the measurements were made.

**Table 2:** In vitro antioxidant and ACEI (angiotensin converting enzyme inhibition) activities of the mousse samples *.

Property	Series I (100% Guar Gum)					Series II (50% Guar Gum: 50% Pectin)				
	Percent of Cow’s Milk Replaced by Whey Protein Hydrolysate (WPH)					Percent of Cow’s Milk Replaced by WPH				
	0	25	50	75	100	0	25	50	75	100
AOX **, μM TE/g of protein	198.5	219.5	219.5	234.1	247.4	214.8	231.4	232.7	232.9	238.4
ACEI activity (IC_50_ ***), mg of protein/mL	19.06	15.85	12.02	10.0	7.56	23.99	6.92	6.03	3.98	2.69

* Throughout the table, SDs (standard deviations) do not exceed 10%. ** AOX—antioxidant activity expressed as equivalents of Trolox (TE).*** the concentration at which ACE activity decreased by 50%.
